# Transcriptional profiling of hepatocytes infected with the replicative form of the malaria parasite *Plasmodium cynomolgi*

**DOI:** 10.1186/s12936-022-04411-3

**Published:** 2022-12-23

**Authors:** Gabriel Mitchell, Guglielmo Roma, Annemarie Voorberg-van der Wel, Martin Beibel, Anne-Marie Zeeman, Sven Schuierer, Laura Torres, Erika L. Flannery, Clemens H. M. Kocken, Sebastian A. Mikolajczak, Thierry T. Diagana

**Affiliations:** 1 Open Innovation at Novartis Institute for Tropical Diseases, Novartis Institutes for BioMedical Research, Emeryville, CA USA; 2 Chemical Biology & Therapeutics, Novartis Institutes for BioMedical Research, Basel, Switzerland; 3 Department of Parasitology, Biomedical Primate Research Centre, Rijswijk, The Netherlands; 4 Novartis Institute for Tropical Diseases, Novartis Institutes for BioMedical Research, Emeryville, CA USA

**Keywords:** Host response, Relapsing malaria, Schizonts, Hypnozoites, RNA sequencing, Liver cells, Monkey

## Abstract

**Background:**

The zoonotic simian parasite *Plasmodium cynomolgi* develops into replicating schizonts and dormant hypnozoites during the infection of hepatocytes and is used as a model organism to study relapsing malaria. The transcriptional profiling of *P. cynomolgi* liver stages was previously reported and revealed many important biological features of the parasite but left out the host response to malaria infection.

**Methods:**

Previously published RNA sequencing data were used to quantify the expression of host genes in rhesus macaque hepatocytes infected with *P. cynomolgi* in comparison to either cells from uninfected samples or uninfected bystander cells.

**Results:**

Although the dataset could not be used to resolve the transcriptional profile of hypnozoite-infected hepatocytes, it provided a snapshot of the host response to liver stage schizonts at 9–10 day post-infection and identified specific host pathways that are modulated during the exo-erythrocytic stage of *P. cynomolgi*.

**Conclusions:**

This study constitutes a valuable resource characterizing the hepatocyte response to *P. cynomolgi* infection and provides a framework to build on future research that aims at understanding hepatocyte-parasite interactions during relapsing malaria infection.

**Supplementary Information:**

The online version contains supplementary material available at 10.1186/s12936-022-04411-3.

## Background

The protozoan parasites *Plasmodium falciparum* and *Plasmodium vivax* are the main sources of human malaria, a disease that globally impacts hundreds of millions of lives each year [1]. The life cycle of malaria includes an asymptomatic liver stage of infection during which the genome from a single parasite is amplified thousands of times in a process known as schizogony, heavily relying on interactions with the host cell [2]. In addition to form replicative liver stage schizonts, *P. vivax* and other relapsing malaria species can differentiate into dormant hypnozoites that can reactivate months or even years after the initial infection, and for which only limited treatment options are available [3]. A better understanding of the host processes required during the liver stages of malaria might lead to the identification of targets for the development of host-directed therapies [4]. Host-directed therapies are especially relevant to hypnozoites considering the tolerance of non-replicating microbes to conventional antimicrobials [5].

Efforts to understand the biology of hypnozoites and to develop radical cure treatments against malaria are dampened by major technical challenges, some of which relate to inherent characteristics of *P. vivax*. In this regard, the zoonotic simian parasite *Plasmodium cynomolgi* [6] has been used as a model organism to study relapsing malaria and the biology of liver stage hypnozoites [3, 7]. Using FACS-purification of hepatocytes infected with GFP-expressing transgenic parasites, the transcriptional profiles of *P. cynomolgi* liver stages was previously reported and revealed many important biological features of the parasite but did not investigate the host response to malaria infection [8–10]. In this follow-up study, previously published RNA sequencing data were re-analysed to define the transcriptional signature of cultured rhesus macaque hepatocytes infected with *P. cynomolgi*.

## Methods

### Ethics statement

Nonhuman primates were used because no other models (in vitro or in vivo) were suitable for the aims of this project. The research protocol was approved by the local independent ethical committee conform Dutch law (BPRC Dier Experimenten Commissie, DEC, agreement number #708). Details were previously described by Voorberg-van der Wel et al. [9].

### Preparation of samples

Naive cells from uninfected samples and uninfected bystander (GFP-negative, negative) cells were isolated and processed for RNA sequencing in-parallel with previously published *P. cynomolgi*-infected samples [9, 10]. The transgenic *P. cynomolgi* M strain PcyC-PAC-GFP_hsp70_-mCherry_ef1α_ [11] as well as the procedures for sporozoite production, liver cell isolation and cell sorting [9, 11] were previously described in detail [12]. Briefly, sporozoites were added to isolated hepatocytes and cultured for 6–10 days before trypsin harvesting. Cells were then sorted using a BD FACS Aria flow cytometer, and fractions were collected in Trizol and stored at − 80 °C until RNA extraction [9–12]. Primary hepatocytes isolated from the same source were used for any given experiment to ascertain that any heterogeneity in the cell population was represented similarly in each sample. Although the contamination of the uninfected bystander cells by unproperly sorted infected cells cannot be completely excluded, this contamination should have been, at worst, infrequent considering the low infection rate associated with the liver stages of malaria [3] and the FACS-based cell sorting protocol used in this study. Accordingly, 99.78% of the reads that mapped to either the parasite or the host genome were from host origin in the non-excluded GFP-negative samples (Additional file 1; See Processing and normalization of RNA sequencing data). Naive cells from uninfected samples were treated similarly to other samples and were FACS-sorted but were not in contact with salivary gland-derived material from uninfected mosquitoes.

### Processing and normalization of RNA sequencing data

The 76 bp paired-end reads were aligned to the reference rhesus monkey genome rheMac7 [13] and used for gene expression quantification with the Exon Quantification Pipeline (EQP) [14], as described by Voorberg-van der Wel et al. [9]. The reference rhesus monkey genome rheMac7 was previously annotated with human gene identification numbers [13]. Gene raw counts were log transformed, normalized and a differential gene expression analysis was performed using the limma/voom Bioconductor pipeline [15] (EdgeR version 3.28.1, limma version 3.42.2) and R (version 3.6.0, 2019-04-26) using a x86_64-pc-linux-gnu (64-bit) platform running under a CentOS Linux 7 (Core). Gene expression analyses were not paired.

### Sample distances, clustering and multidimensional scaling analyses

Sample distances and clustering were calculated using the Poisson distance [16], the gene raw counts and RStudio (version 1.1.456). Poisson distances were also used to generate multidimensional scaling (MDS) plots in RStudio, as described by Love et al. [16].

### Pathway enrichment and Venn diagram analyses

Pathway enrichment analyses were performed by filtering the differentially expressed genes using a threshold for statistical significance (absolute log_2_ fold change > 1 and adjusted *P*_value_ < 0.05) and Metascape [17]. GSEAs were performed using GSEABase (version 1.48.0) and gene sets from the MSigDB collections (version 6.2) [18–20]. Pathway enrichment analyses were performed against all annotated human genes available on Metascape and GSEABase. Venn diagram analyses were performed using the tool provided on the Bioinformatics & Evolutionary Genomics website of Ghent University [21].

### Preparation of graphs and statistical analyses

Graphs were generated using Metascape [17], RStudio (version 1.1.456) and the GraphPad Prism software (v.9.2.0). The use of *P*_values_ adjusted for the False Discovery Rate (FDR) (adjusted *P*_values_) was favoured throughout this study to correct for multiple comparisons and decrease the occurrence of false positives.

## Results

### Transcriptomic analysis of primary hepatocytes infected with *P. cynomolgi* schizonts

To gain insight into the host response during the liver stages of malaria, previously published RNA sequencing datasets obtained from primary simian hepatocytes infected with *P. cynomolgi* [9, 10] were aligned to a reference host genome and used to quantify the expression of host genes in comparison to naive cells from uninfected samples (see Methods). More precisely, *P. cynomolgi*-infected hepatocytes were cultured for 6–10 days and subsequently FACS-purified, yielding GFP-expressing hypnozoites (GFP-low) and schizonts (GFP-high). These samples were then compared to naive uninfected cells using RNAseq. Preliminary results showed that while samples infected for 6–7 days were associated with only few statistically significant differentially expressed genes, samples infected for 9–10 days were associated with numerous statistically significant changes in comparison to uninfected samples (data not shown). As GFP-low samples were previously shown to be contaminated with uninfected cells as well as with schizont or released merozoite transcripts at 9–10 day post-infection (dpi) [10], the GFP-low samples were excluded from this study (See Additional file 2 for a descriptive list of samples used in this study) and the hepatocyte response to hypnozoites was not determined. Despite this, the 9–10 dpi GFP-high samples were further analysed to provide a snapshot of the transcriptional response of primary rhesus macaque hepatocytes to *P. cynomolgi* schizonts.

**Fig. 1:**
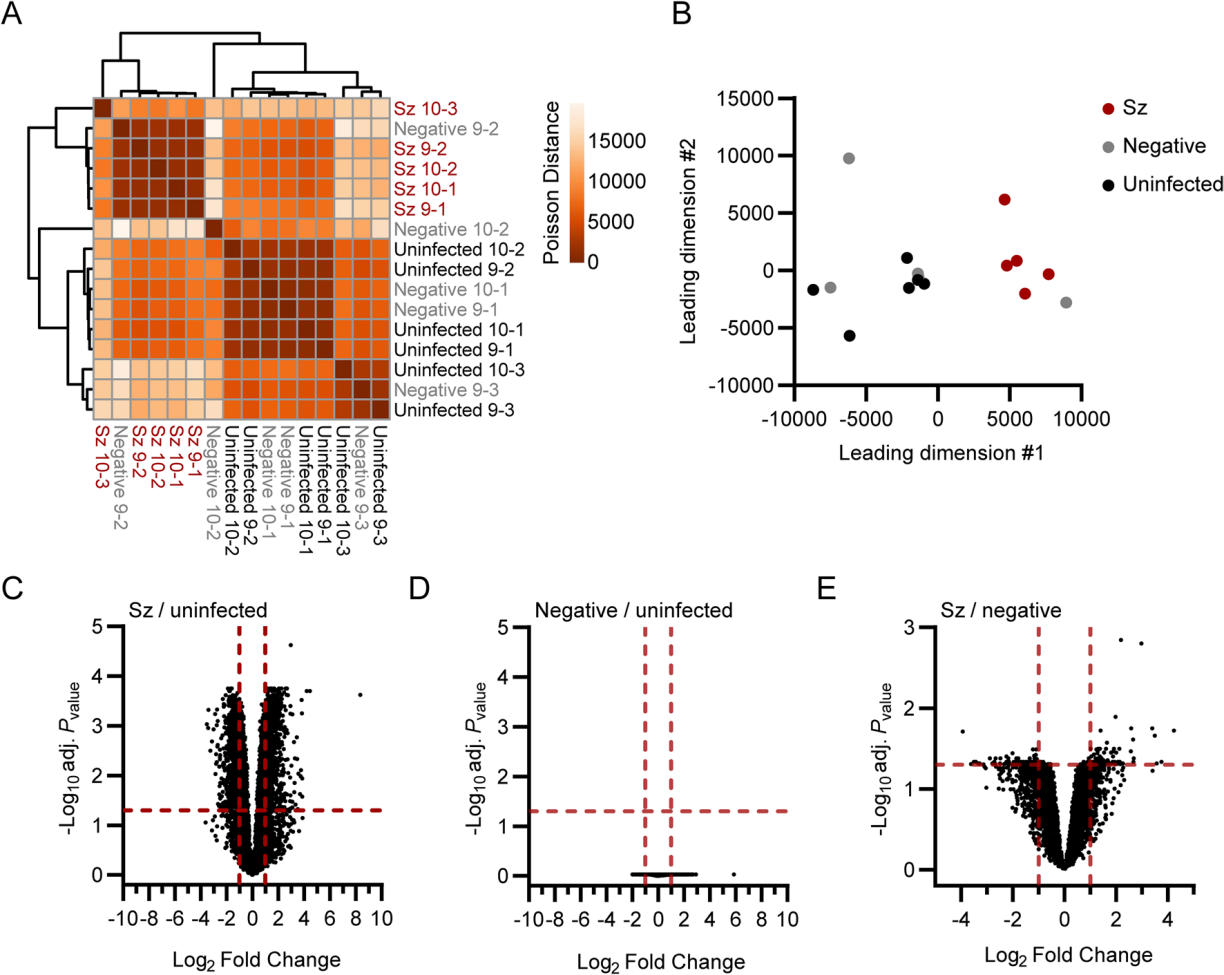
Transcriptomic analysis of primary rhesus macaque hepatocytes infected with *P. cynomolgi* schizonts*. *Heat map (**A**) and multidimensional scaling (MDS) plot (**B**) showing sample-to-sample separation of cells infected with schizonts (Sz, in red), uninfected bystander (Negative, in grey) cells and cells from uninfected samples (Uninfected, in black). Volcano plots showing mean log_2_ fold changes and -log_10_ adjusted *P*_values_ (adj. *P*_value_) for cells infected with schizonts in comparison to uninfected samples (**C**), negative cells in comparison to uninfected samples (**D**) and cells infected with schizonts in comparison to negative cells (**E**). Horizontal and vertical dotted red lines indicate adjusted *P*_values_ less than 0.05 [or − log10 (adj. *P*_value_) greater than 1.3] and absolute log_2_ fold changes greater than 1, respectively

Global transcriptional differences between schizont-infected cells, uninfected bystander (GFP-negative, negative) cells and naive cells from uninfected samples were evaluated using sample-to-sample distance analyses (Fig. 1A, B). While clusters for schizont-infected cells and cells from uninfected samples were clearly separated in space, the clustering of the negative samples were not as defined. This variation might be of either technical or biological origins and might have been influenced by a heterogeneous response to salivary gland-derived material or by a heterogeneous response of bystander uninfected cells to infection. However, the GFP-negative samples tended to be distributed closer to the uninfected than the schizont-infected samples, suggesting that the transcriptional profile of schizont-infected cells was mainly a direct result of the infection. It was reasoned that while the comparison of schizont-infected cells to uninfected samples might reveal a larger number of statistically significant gene modulations, the comparison of schizont-infected cells to uninfected bystander cells was likely to be more stringent and reveal genes specifically modulated in schizont-infected hepatocytes. Therefore, the transcriptional response of schizont-infected cells was determined in comparison to both uninfected samples and uninfected bystander cells, allowing the identification of specific signatures with higher confidence.

Thousands of genes were found to be significantly modulated (i.e., genes associated with absolute log_2_ fold change > 1 and adjusted *P*_value_ < 0.05) in schizont-infected hepatocytes in comparison to uninfected samples (Fig. 1C and Additional file 3). No statistically significant changes in gene expression were detected in uninfected bystander (negative) cells in comparison to uninfected samples using the *P*_values_ adjusted for multiple comparisons (Fig. 1D), again suggesting that the transcriptional modulations detected in schizont-infected cells are a direct response to intracellular parasites. However, it is noteworthy that transcriptional changes could be detected in uninfected bystander cells vs uninfected samples using the more permissive standard *P*_values_ (Additional file 4). Moreover, several of the most modulated transcripts in schizont-infected cells were also modulated, although to a lesser extent and not significantly, in negative cells in comparison to uninfected samples (Additional file 5). Relatively fewer transcripts were associated with statistically significant changes in schizont-infected vs. negative cells (Fig. 1E and Additional file 6). Interestingly, transcripts modulated in schizont-infected cells vs. uninfected samples and schizont-infected cells vs. negative cells showed some overlap for the 25 most upregulated (e.g., CXCL19, ADH7 and RGS1) and downregulated (e.g., HOXB9, RNF165 and LGALS7) genes (Additional files 5, 7). Overall, differentially expressed genes were detected in schizont-infected hepatocytes in comparison to both uninfected samples and uninfected bystander cells, but no significant modulations were detected in uninfected bystander cells in comparison to cells from uninfected samples.

### Pathway analysis of differentially expressed genes in schizont-infected hepatocytes in comparison to cells from uninfected samples

**Fig. 2:**
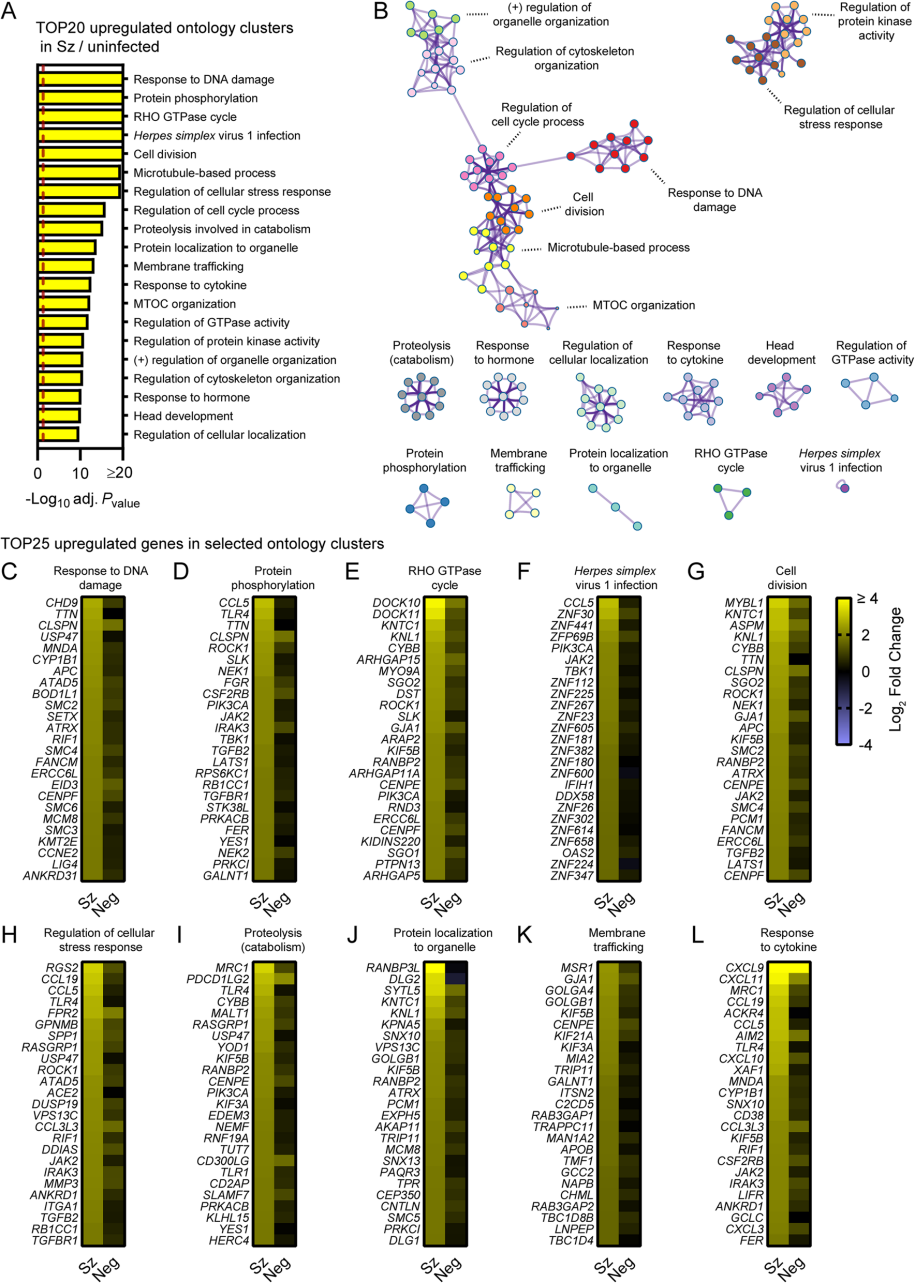
Pathway analysis of genes significantly upregulated in schizont-infected hepatocytes in comparison to uninfected samples. **A** Most enriched ontology clusters for genes significantly upregulated in primary rhesus macaque hepatocytes infected with *P. cynomolgi* schizonts. The vertical dotted red line indicates adjusted *P*_values_ less than 0.05 (or − log10 (adj. *P*_value_) greater than 1.3). The most significantly enriched term of each ontology clusters was selected as the cluster label. **B** Subset of representative terms were selected to generate a network of enriched ontology clusters. Terms are represented by circle nodes with colors that shows belonging to a specific cluster. Circles nodes with similarity scores > 0.3 are linked with edges. **C**–**L** Heat maps of log_2_ fold changes for the 25 most upregulated genes for selected ontology clusters. Data are shown for schizont-infected and uninfected bystander (negative) cells in comparison to uninfected samples. Some annotation terms are abbreviated or modified for a purpose of presentation

To focus on the most robustly modulated genes, filtered-by-threshold pathway enrichment analyses were performed using genes associated with statistically significant changes in schizont-infected cells versus uninfected samples (Fig. 1C and Additional file 3) and Metascape [17]. The 20 most enriched Metascape ontology clusters in genes upregulated by schizont-infected cells (Additional file 8) are represented in a histogram (Fig. 2A) and in a network layout that connects enriched terms with a certain level of similarity (Fig. 2B). Interestingly, 7 of the most significantly enriched ontology clusters were interconnected and shared genes associated with the response to DNA damage, cell division, microtubule-based process, regulation of cell cycle, microtubule-organizing center (MTOC), regulation of cytoskeleton organization and regulation of organelle organization. Two other ontology clusters were also interconnected and included genes involved in regulation of the cellular stress response and in regulation of protein kinase activity. The remaining most significantly enriched ontology clusters were discrete and related to other pathways such as RHO GTPase cycle, response to *Herpes simplex* virus 1 (HSV-1) infection, proteolysis involved in catabolism, membrane trafficking and response to cytokines. Ten ontology clusters were further selected and the expression levels of the 25 most up-regulated genes were visualized for each of these clusters in both schizont-infected and uninfected bystander (negative) cells in comparison to uninfected cells (Fig. 2C–L). While most groups of genes were more strongly and homogenously upregulated in schizont-infected cells, several genes were also expressed at noticeable levels in negative cells (e.g., CLSPN, PDCD1LG2 and CXCL9). The results thus suggested that schizont-infected hepatocytes upregulate multiple biological pathways in comparison to cells from uninfected samples and, in most cases but not always exclusively, in comparison to uninfected bystander cells.

**Fig. 3:**
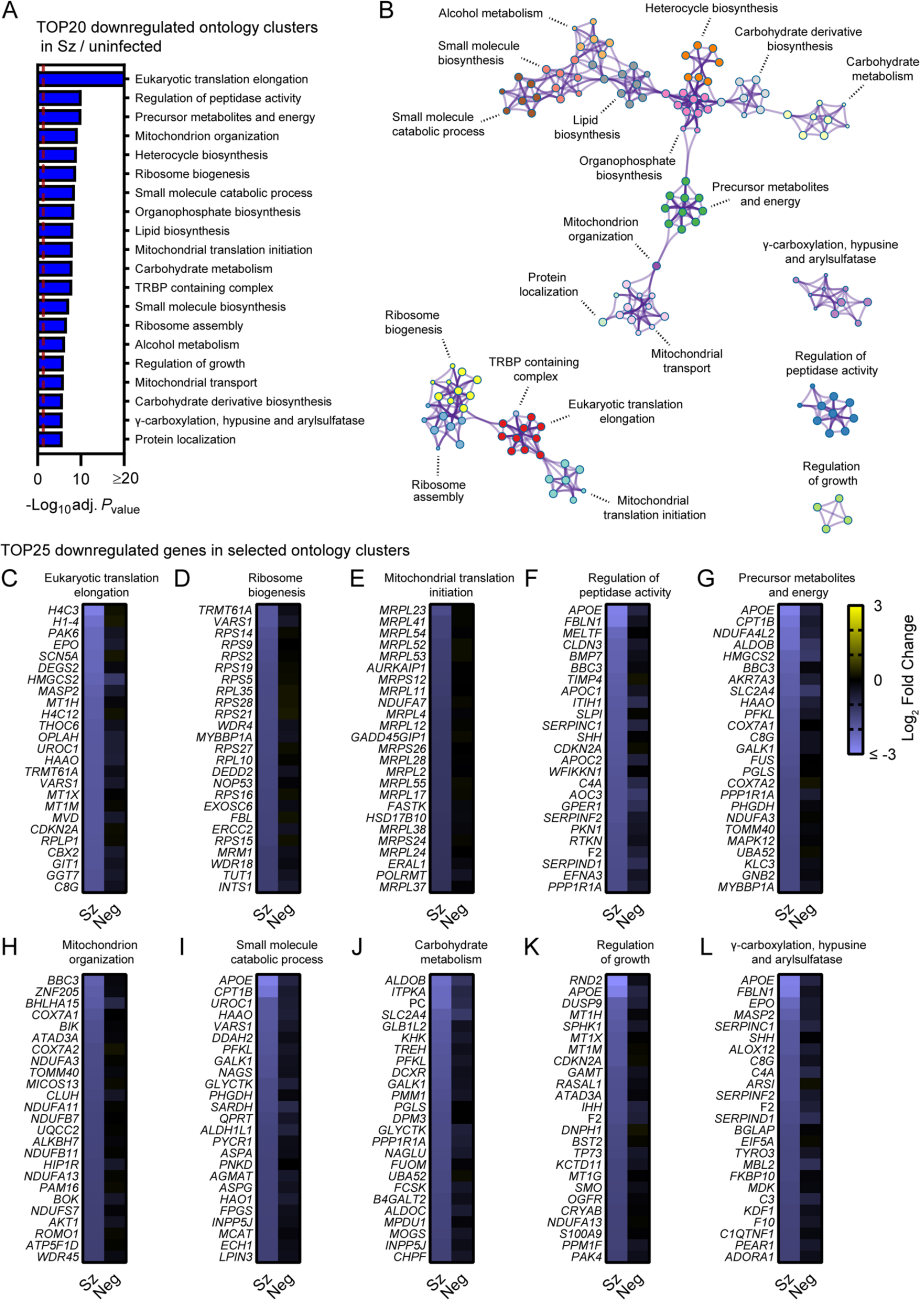
Pathway analysis of genes significantly downregulated in schizont-infected hepatocytes in comparison to uninfected samples. (**A**) Most enriched ontology clusters for genes significantly downregulated in primary rhesus macaque hepatocytes infected with *P. cynomolgi* schizonts. The vertical dotted red line indicates adjusted *P*_values_ less than 0.05 (or − log10 (adj. *P*_value_) greater than 1.3). The most significantly enriched term of each ontology clusters was selected as the cluster label. (**B**) Subset of representative terms were selected to generate a network of enriched ontology clusters. Terms are represented by circle nodes with colors that shows belonging to a specific cluster. Circles nodes with similarity scores > 0.3 are linked with edges. (**C**–**L**) Heat maps of log_2_ fold changes for the 25 most downregulated genes of selected ontology clusters. Data are shown for schizont-infected and uninfected bystander (negative) cells in comparison to uninfected samples. Some annotation terms are abbreviated or modified for a purpose of presentation

Metascape pathway analysis of genes significantly downregulated in schizont-infected cells vs. uninfected samples showed the enrichment of two networks of ontology clusters that relate to protein translation and ribosomes, and metabolic pathways and mitochondria, respectively (Fig. 3A, B, Additional file 9). Three other ontology clusters were distinct and included genes associated with γ-carboxylation, hypusine formation and arylsulfatase activation, regulation of peptidase activity and regulation of growth (Fig. 3B). Interestingly, Metascape analyses also revealed that genes associated with the specific transcriptional signature of the liver tissue (PaGenBase [22]) were significantly enriched among transcripts downregulated in schizont-infected cells (Additional file 10). Analysis of the 25 most down-regulated genes for selected ontology clusters showed robust and overall consistent gene repression in schizont-infected cells in comparison to both uninfected samples and uninfected bystander cells (Fig. 3C–L). These results suggested that schizont-infected hepatocytes significantly downregulate genes involved in multiple biological pathways, including pathways associated with protein translation and metabolic processes, as well as genes associated with the transcriptional signature of the liver tissue.

### Pathway analysis of differentially expressed genes in schizont-infected hepatocytes in comparison to uninfected bystander cells

**Fig. 4:**
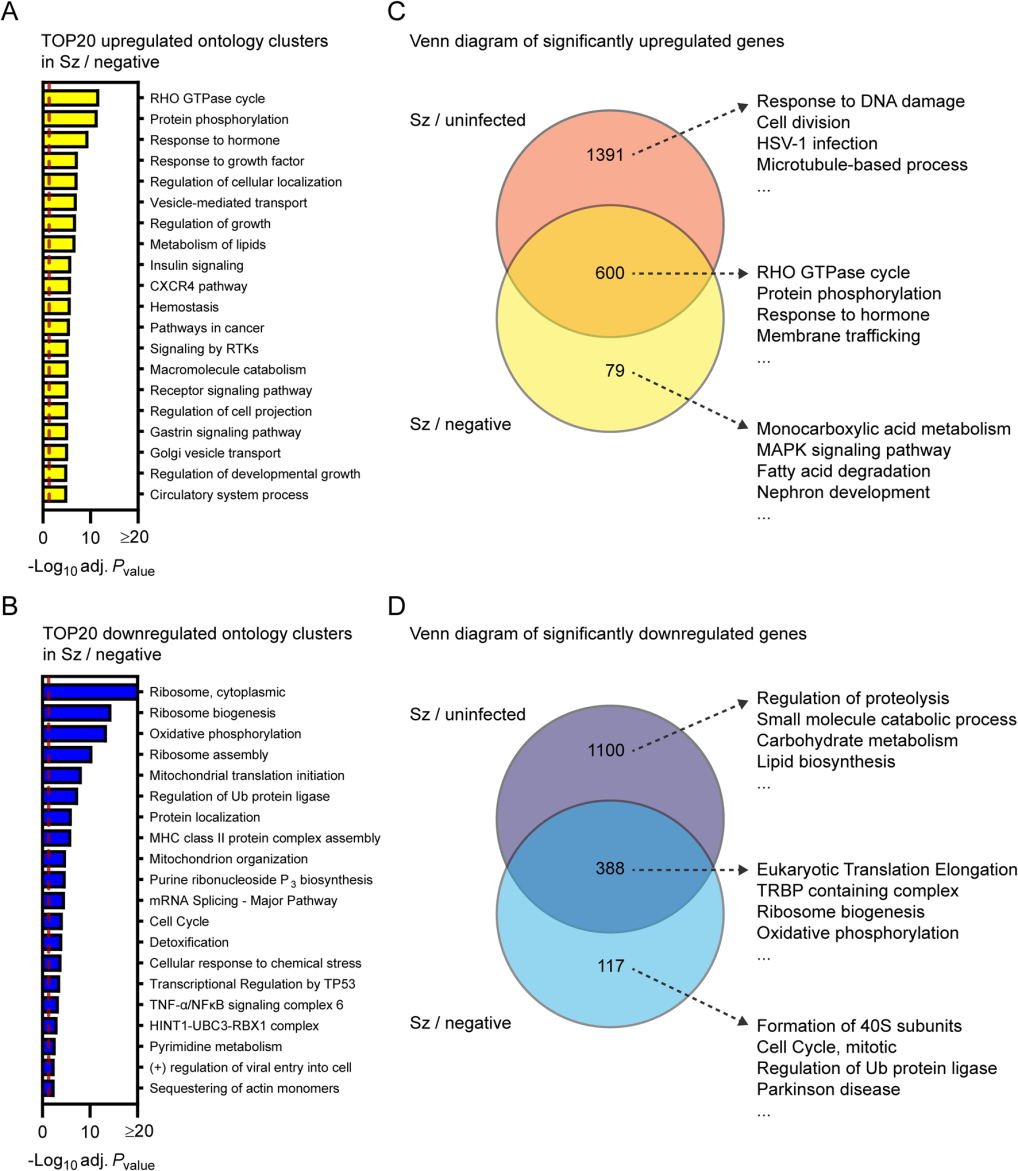
Transcriptional response of schizont-infected hepatocytes in comparison to uninfected bystander cells. Most enriched ontology clusters for genes significantly upregulated (**A**) and downregulated (**B**) in hepatocytes infected with schizonts in comparison to uninfected bystander (negative) cells. Venn diagram analyses comparing upregulated (**C**) and downregulated (**D**) genes in schizont-infected hepatocytes vs. uninfected samples or in schizont-infected hepatocytes vs. negative cells. The 4 most enriched ontology clusters are shown for each subgroup of genes. Dotted red lines indicate adjusted *P*_values_ less than 0.05 (or − log10 (adj. *P*_value_) greater than 1.3). Some annotation terms are abbreviated or modified for a purpose of presentation

To confirm results from the previous section as well as to identify transcriptional changes strictly modulated in infected cells, Metascape pathway analysis was performed for the transcripts significantly modulated in schizont-infected vs. uninfected bystander (negative) cells. This analysis revealed enrichment of ontology clusters associated with specific biological processes in both upregulated (e.g., RHO GTPase cycle and protein phosphorylation) (Fig. 4A and Additional file 11) and downregulated (e.g., ribosome and oxidative phosphorylation) genes (Fig. 4B and Additional file 11). This analysis also confirmed the downregulation of genes associated with the liver-specific transcriptional signature in *P. cynomolgi*-infected hepatocytes (Additional file 12).

Venn diagram analysis was used to compare the differentially expressed genes in schizont-infected hepatocytes vs. cells from uninfected samples and in schizont-infected hepatocytes vs. negative cells, and the genes of each subgroup were then submitted to a Metascape pathway analysis (Fig. 4C, D). Genes only upregulated in schizont-infected cells vs. uninfected samples were associated with specific ontology clusters, such as response to DNA damage, cell division, HSV-1 infection, and microtubule-based process (Fig. 4C and Additional file 13) and might either represent genes that are also induced in the uninfected bystander (negative) cells or more sensitive to noise (i.e., and more impacted by the greater variability between negative samples in comparison to uninfected samples). Genes upregulated in both schizont-infected cells vs. uninfected samples and schizont-infected cells vs. negative cells were associated with other ontology clusters, such as Rho GTPase cycle, protein phosphorylation, response to hormone and membrane trafficking (Fig. 4C and Additional file 13), and represent transcripts specifically induced in infected cells. Results similarly show the specific enrichment of ontology clusters for genes only downregulated in schizont-infected vs. uninfected samples (e.g., regulation of proteolysis and small molecule catabolic process) or downregulated in both schizont-infected vs. uninfected samples and schizont-infected vs. negative cells (e.g., eukaryotic translation elongation, TRBP containing complex, ribosome biogenesis and oxidative phosphorylation) (Fig. 4D and Additional file 14). Ontology clusters enriched in genes specifically modulated by schizont-infected cells vs. negative cells were more difficult to interpret but might reflect changes induced by the infection in the uninfected bystander cell population (Fig. 4C, D). Altogether, these results showed that changes in different biological processes are detected in schizont-infected hepatocytes depending on whether the data are compared to naive cells from uninfected samples or to uninfected bystander cells.

It was hypothesized that differences in the transcriptional profiles of samples normalized to either uninfected samples or negative cells were caused by the modulation of specific transcripts, although below our threshold for statistical significance (absolute log_2_ fold change > 1 and adjusted *P*_value_ < 0.05), in the uninfected bystander (negative) cell population. Accordingly, numerous transcripts were differentially expressed in negative cells in comparison to uninfected samples once the data were filtered using a more permissive threshold (e.g., absolute log_2_ fold change > 1 and *P*_value_ < 0.05) (Additional file 4). To better understand the transcriptional changes in negative cells, data were analyzed using Gene Set Enrichment Analysis (GSEA) (Additional file 15), a threshold-free method that does not require prior filtering of genes based on statistically significant transcriptional changes [18–20]. Comparison of GSEA pathways enriched in schizont-infected cells and negative cells using all gene sets from the MSigDB collection showed a considerable overlap for both the upregulated and, to a lesser extent, downregulated pathways (Additional file 16). Interestingly, among selected gene sets (i.e., Gene Ontology (GO), the KEGG pathway and the Reactome pathway databases), GSEA pathways that relate to chromosome biology were significantly enriched in both schizont-infected and negative cells in comparison to uninfected samples, but not in schizont-infected vs. negative cells (Additional file 17), demonstrating a common regulation of these pathways in infected and uninfected bystander cells. Overall, these results strongly suggested that a group of specific host processes are modulated at the transcriptional level in the uninfected bystander cell population. However, whether or not these differences are of technical or biological origins is unknown. Importantly, it is possible that these differences between the naive uninfected cells and the uninfected bystander cells are influenced by both the hepatocyte response to salivary gland-derived material and the indirect response of uninfected bystander cells to malaria infection.

### Host transcriptional profiling of the liver stage of *Plasmodium berghei*

A dual RNA sequencing study for the liver stage of the non-relapsing rodent malaria parasite *P. berghei* was previously published [23]. To compare the data more directly to this study, the combined host gene expression dataset for hepatocyte-like cell lines (Huh7.5.1, HC04 and HepG2) infected with *P. berghei* for 48 h (See Supplementary Data 5 published by LaMonte et al. [23]) was re-analysed with the filtered-by-threshold approach used in this study (i.e., absolute log_2_ fold change > 1 and adjusted *P*_value_ < 0.05) and Metascape. Similarly to *P. cynomolgi*-infected hepatocytes, cells infected with *P. berghei* upregulated genes associated with *Herpes simplex* virus 1 (HSV-1) infection, microtubule-based process and RHO GTPase cycle while downregulating genes associated with translation, oxidative phosphorylation and metabolic processes (Additional file 18). In addition, genes associated with the specific transcriptional signature of the liver tissue (PaGenBase database) were also significantly enriched among transcripts downregulated in *P. berghei*-infected cells (Additional file 19). Thus, the transcriptomes of cells infected with *P. cynomolgi* and *P. berghei* liver stage schizonts show modulation of similar biological processes.

## Discussion

This study provides a snapshot of the transcriptional profile of primary rhesus macaque hepatocytes infected with the replicative form of the malaria parasite *P. cynomolgi*. It was found that schizont-infected cells modulate the expression of genes associated with multiple host processes in comparison to naive cells from uninfected samples but also to uninfected bystander cells. The reason why some gene modulations were only observed in schizont-infected hepatocytes when normalized to uninfected samples but not to uninfected bystander cells is not known, and might be of technical (e.g., greater variability within the uninfected bystander cell population) or of biological (e.g., heterogeneous induction of genes in bystander cells) origin. However, while the comparison to uninfected samples provides a more exhaustive picture of the host response to infection, the differentially expressed genes identified from the comparison to uninfected bystander cells are more likely to be specific to schizont-infected hepatocytes.

The host pathways modulated in cells infected with the liver stages of *P. cynomolgi* and *P. berghei* considerably overlap. Similarly to *P. cynomolgi*-infected hepatocytes, our re-analysis of the dual RNAseq dataset previously published by LaMonte et al. [23] showed that cells infected with the liver stage of *P. berghei* upregulate genes associated with *Herpes simplex* virus 1 (HSV-1) infection, microtubule-based processes and the RHO GTPase cycle (Additional file 18), and down-regulate genes associated with translation, oxidative phosphorylation and liver-specific functions (Additional files 18, 19). The upregulation of genes associated with HSV-1 infection and the response to cytokines in hepatocytes infected with *P. cynomolgi* schizonts (Fig. 2) also agrees with a previous study suggesting that *P. berghei* activates innate immunity during the liver stage of infection [24]. However, as strongly suggested by another study profiling the host transcriptome during the liver stage of *P. berghei* [25], the response to infection is not only dynamic and evolved as a function of time but is also highly dependent on the background of host cells being infected. Consequently, differences between the response of primary rhesus macaque hepatocytes and immortalized hepatocyte-like human cells to malaria parasites might be exacerbated by their respective proliferative state as well as by species-specific variations. The host pathways that are modulated independently of the host and parasite species might represent the specific transcriptional signature of schizont-infected hepatocytes and are likely to include host processes that are essential for liver stage development.

Recent studies reported the host transcriptional profile of human hepatocytes infected with *P. vivax* [26, 27]. Interestingly, Mancio-Silva et al. [26] found that the late phase of the *P. vivax* liver stage is associated with the downregulation of liver-specific functions as well as translation, as observed for *P. cynomolgi*- and *P. berghei*-infected hepatocytes (Figs. 3, 4 and Additional files 10, 12, 18, 19). However, few significantly upregulated genes were observed for *P. vivax*-infected hepatocytes [26], contrasting with the potent induction of numerous host transcripts during the liver stages of *P. berghei* [23] and *P. cynomolgi* (Fig. 1). Moreover, the study by Ruberto et al. [27] reported opposite modulations of host pathways (e.g., upregulations of host genes associated with translation and oxidative phosphorylation) in hepatocytes infected with *P. vivax*, which is in apparent disagreement with our results, as well as with results from others [23, 26]. Thus, beyond the impact of malaria- and host-species specific variations, current results suggest that the hepatocyte response to infection is highly dynamic or influenced by technical aspects (e.g., the source of human hepatocytes). Accordingly, more thorough studies are required to accurately compare the transcriptome of hepatocytes infected by different species of malaria.

The lack of data for the transcriptional profiling of hepatocytes infected with *P. cynomolgi* hypnozoites is a major limitation of this study. This limitation was caused by the technical challenge of sorting hypnozoite-infected hepatocytes at later infection timepoints using the FACS-based approach [10]. This study also only provides a snapshot of the transcriptional signature of schizont-infected cells at 9–10 days post-infection. As such, future transcriptomic studies should be performed at different timepoints to evaluate the dynamic aspect of the host response as well as to compare the host transcriptional signature of hepatocytes infected with schizonts and hypnozoites. Moreover, this study did not identify any significantly modulated genes in the bystander uninfected cell population (Fig. 1), which might be caused by a heterogeneity in the transcriptome of these cells and could be better resolved using spatial transcriptomic technologies [28].

## Conclusions

Despite its limitations, this pioneering study characterized the transcriptional signature of rhesus macaque hepatocytes infected with the replicative form of *P. cynomolgi* and found similitudes with the host response induced by the liver stages of other malaria species. This study thus further validates *P. cynomolgi* as a model organism to study relapsing malaria and provides a framework to build on future research that aims at understanding host–pathogen interactions during the liver stages of malaria infection.

## Supplementary Information

**Additional file 1.** Host read counts and host expressed read counts for samples used in this study. Additional file table.

**Additional file 2.** Samples used in this study. Additional file table.

**Additional file 3.** Transcriptomic analysis of schizont-infected and uninfected bystander (negative) primary rhesus macaque hepatocytes in comparison to uninfected samples. This file includes read counts, fold changes, *P* and adjusted *P* values as well as lists of genes significantly up- or down-regulated. Source data associated with Fig. 1.

**Additional file 4.** Volcano plots showing mean log_2_ fold changes and − log_10_ *P*_values_ for schizont-infected vs. uninfected samples (**A**), uninfected bystander (negative) cells vs uninfected samples (**B**) and schizont-infected vs. negative cells (**C**). Additional file figure.

**Additional file 5.** Most up- and down-regulated genes in primary rhesus macaque hepatocytes infected with *P. cynomolgi* schizonts in comparison to uninfected samples. Supplemental table associated with Fig. 1.

**Additional file 6.** Transcriptomic analysis of primary rhesus macaque hepatocytes infected with schizonts in comparison to uninfected bystander (negative) cells. This file includes fold changes, *P* and adjusted *P*_values_ as well as lists of genes significantly up- or down-regulated. Source data associated with Fig. 1.

**Additional file 7.** Most up- and down-regulated genes in primary rhesus macaque hepatocytes infected with *P. cynomolgi* schizonts in comparison to uninfected bystander (negative) cells. Supplemental table associated with Fig. 1.

**Additional file 8.** Term annotation and pathway enrichment analysis for genes significantly upregulated in primary rhesus macaque hepatocytes infected with schizonts in comparison to uninfected samples. This analysis was performed with Metascape. Source data associated with Fig. 2.

**Additional file 9.** Term annotation and pathway enrichment analysis for genes significantly downregulated in primary rhesus macaque hepatocytes infected with schizonts in comparison to uninfected samples. This analysis was performed with Metascape. Source data associated with Fig. 3.

**Additional file 10.** Most enriched PaGenBase (Pattern Gene Database) terms for genes significantly downregulated in primary rhesus macaque hepatocytes infected with schizonts in comparison to uninfected samples. The vertical dotted red line indicates adjusted *P*_values_ less than 0.05 (or − log10 (adj. *P*_value_) greater than 1.3). This analysis was performed with Metascape. Additional figure associated with Fig. 3.

**Additional file 11.** Term annotation and pathway enrichment analysis for genes significantly modulated in primary rhesus macaque hepatocytes infected with schizonts in comparison to uninfected bystander (negative) cells. This analysis was performed with Metascape. Source data associated with Fig. 4.

**Additional file 12.** Most enriched PaGenBase (Pattern Gene Database) terms for genes significantly downregulated in primary rhesus macaque hepatocytes infected with schizonts in comparison to uninfected bystander (negative) cells. The vertical dotted red line indicates adjusted *P*_values_ less than 0.05 (or − log10 (adj. *P*_value_) greater than 1.3). This analysis was performed with Metascape. Supplemental figure associated with Fig. 4.

**Additional file 13.** Venn diagram analysis lists, term annotation and Metascape pathway enrichment analysis for significantly upregulated genes in primary rhesus macaque hepatocytes infected with schizonts in comparison to uninfected samples or uninfected bystander (negative) cells. Source data associated with Fig. 4.

**Additional file 14.** Venn diagram analysis lists, term annotation and Metascape pathway enrichment analysis for significantly downregulated genes in primary rhesus macaque hepatocytes infected with schizonts in comparison to uninfected samples or uninfected bystander (negative) cells. Source data associated with Fig. 4.

**Additional file 15.** Gene Set Enrichment Analysis (GSEA) for the transcriptional profiling of schizont-infected cells vs. uninfected samples, uninfected bystander (negative) cells vs. uninfected samples and schizont-infected cells vs. negative cells. This file includes lists for all and selected gene sets (i.e., Gene Ontology (GO), the KEGG pathway and the Reactome pathway databases) from the MSigDB collections. Only datasets associated with a False Discovery Rate (FDR) < 0.05 are shown. Additional source data.

**Additional file 16.** Venn diagram analyses comparing upregulated (**A**) and downregulated (**B**) gene sets / pathways from GSEA analyses in schizont-infected vs. uninfected samples and uninfected bystander (negative) cells vs. uninfected samples. All gene sets from the MSigDB collections were considered for these analyses. Additional file figure.

**Additional file 17.** Most significantly enriched gene sets from GSEAs for the upregulated (yellow) and downregulated (blue) transcripts in schizont-infected vs. uninfected samples (**A**), uninfected bystander (negative) cells vs. uninfected samples (**B**) and schizont-infected vs. negative cells (**C**). Only gene sets from the Gene Ontology (GO), the KEGG pathway and the Reactome pathway databases were considered. Some annotation terms are abbreviated or modified for a purpose of presentation. Additional file figure.

**Additional file 18.** Re-analysis of the host response to *P. berghei *liver stage schizonts. Most enriched ontology clusters for genes significantly upregulated (**A**) and downregulated (**B**) in cells infected with *P. berghei* liver stage schizonts. Dotted red lines indicate adjusted *P*_values_ less than 0.05 (or − log10 (adj. *P*_value_) greater than 1.3). Some annotation terms are abbreviated or modified for a purpose of presentation. This analysis was performed with Metascape. Additional file figure.

**Additional file19.** Re-analysis of the host response to *P. berghei *liver stage schizonts. Most enriched PaGenBase (Pattern Gene Database) terms for genes significantly downregulated in cells infected with *P. berghei* liver stage schizonts. The vertical dotted red line indicates adjusted *P*_values_ less than 0.05 (or − log10 (adj. *P*_value_) greater than 1.3). This analysis was performed with Metascape. Additional file figure.

## Data Availability

The raw RNA-sequencing reads for the infected samples analyzed in this study were previously published [10] and made available in the NCBI Short Read Archive (https://www.ncbi.nlm.nih.gov/sra) under accession number SRP096160 (https://www.ncbi.nlm.nih.gov/sra/?term=SRP096160). The raw RNA-sequencing reads for uninfected samples and uninfected bystander (negative) cells are also available in the NCBI Short Read Archive under accession number SRP392210 (https://www.ncbi.nlm.nih.gov/sra?term=SRP392210). Read counts for samples analyzed in this study are also included in Additional file 3.

## Abbreviations

Adj *P*_value_: Adjusted *P*_value_
BP: Base pair
DPI: Day post-infection
EQP: Exon quantification pipeline
FACS: Fluorescence-activated cell sorting
FDR: False discovery rate
GFP: Green fluorescent protein
GFP-neg: GFP-negative
GO: Gene ontology
GSEA: Gene set enrichment analysis
HSV-1: *Herpes simplex* virus 1
Hz: Hypnozoite
ID: Identification
MDS: Multidimensional scaling
MTOC: Microtubule-organizing center
NS: Non-significant
PaGenBase: Pattern gene database
*P. berghei*: *Plasmodium berghei*
*P. cynomolgi*: *Plasmodium cynomolgi*
*P. vivax*: *Plasmodium vivax*
RNA: Ribonucleic acid
RNAseq: RNA sequencing
Sz: Schizont
