# Supplementary material for: Transcriptional profiling of hepatocytes infected with the replicative form of the malaria parasite Plasmodium cynomolgi
Source: Malar J. 2022 Dec 23;21:393. doi: 10.1186/s12936-022-04411-3 (PMC9789591; doi:10.1186/s12936-022-04411-3)
Supplement: Supplementary file 1 — Additional file 1. Host read counts and host expressed read counts for samples used in this study. Additional file table. [file 12936_2022_4411_MOESM1_ESM.docx]

**Additional file 1.** Host read counts and host expressed read counts for samples used in this study*^a^*

| Infection ID | Sample ID | # total reads | # mapped reads*^c^* | # host reads*^d^* | % host reads vs mapped reads^e^ | # expr. host reads *^f^* | % expr. host reads vs host reads*^g^* |
| --- | --- | --- | --- | --- | --- | --- | --- |
|  |  |  |  |  |  |  |  |
| PAC.27.F1 | Sz 9-1 | 66534612 | 54176231 | 21095681 | 38.94 | 17765194 | 84.21 |
|  | Hz 9-1*^b^* | 52110731 | 28376922 | 28217574 | 99.44 | 22174914 | 78.59 |
|  | Negative 9-1 | 57836914 | 44575047 | 44477955 | 99.78 | 36748132 | 82.62 |
|  | Uninfected 9-1 | 62754802 | 47465813 | 47459183 | 99.99 | 39888812 | 84.05 |
|  | Sz 10-1 | 56463837 | 45668706 | 20455117 | 44.79 | 17334498 | 84.74 |
|  | Hz 10-1*^b^* | 58921274 | 47819689 | 46394102 | 97.02 | 39385752 | 84.89 |
|  | Negative 10-1 | 55405362 | 43924342 | 43828466 | 99.78 | 36884621 | 84.16 |
|  | Uninfected 10-1 | 59386138 | 47015654 | 47009785 | 99.99 | 40090399 | 85.28 |
|  |  |  |  |  |  |  |  |
| PAC.27.F2 | Sz 9-2 | 55909042 | 45319535 | 14013282 | 30.92 | 11200792 | 79.93 |
|  | Hz 9-2*^b^* | 61545900 | 50009904 | 49669566 | 99.32 | 42164869 | 84.89 |
|  | Negative 9-2 | 62745994 | 51240093 | 51145241 | 99.81 | 43192038 | 84.45 |
|  | Uninfected 9-2 | 60588923 | 48463468 | 48456411 | 99.99 | 40977430 | 84.57 |
|  | Sz 10-2 | 50290660 | 40665396 | 15168123 | 37.30 | 12891304 | 84.99 |
|  | Hz 10-2*^b^* | 90906552 | 73497272 | 72429345 | 98.55 | 60855237 | 84.02 |
|  | Negative 10-2 | 79819929 | 57105948 | 56912776 | 99.66 | 44481305 | 78.16 |
|  | Uninfected 10-2 | 55837246 | 37752499 | 37744275 | 99.98 | 31021867 | 82.19 |
|  |  |  |  |  |  |  |  |
| PAC.30.F2 | Sz 9-3*^b^* | 10725766 | 2223473 | 1111300 | 49.98 | 242659 | 21.84 |
|  | Hz 9-3*^b^* | 3668867 | 2759915 | 2514510 | 91.11 | 1970549 | 78.37 |
|  | Negative 9-3 | 74370233 | 58448760 | 58386899 | 99.89 | 48571581 | 83.19 |
|  | Uninfected 9-3 | 82662285 | 64870461 | 64860823 | 99.99 | 54537088 | 84.08 |
|  | Sz 10-3 | 47422264 | 38049370 | 15713470 | 41.30 | 12764026 | 81.23 |
|  | Hz 10-3*^b^* | 76764315 | 62233366 | 57730038 | 92.76 | 46512265 | 80.57 |
|  | Negative 10-3*^b^* | 1027021 | 172323 | 167753 | 97.35 | 51421 | 30.65 |
|  | Uninfected 10-3 | 72244844 | 57547074 | 57542299 | 99.99 | 49887419 | 86.70 |
|  |  |  |  |  |  |  |  |
| PAC.31.F1.F2 | Sz 10-4*^b^* | 66921551 | 57486670 | 44834680 | 77.99 | 38048997 | 84.87 |
|  | Hz 10-4*^b^* | 78594602 | 67607100 | 67508002 | 99.85 | 57456052 | 85.11 |
|  | Negative 10-4*^b^* | 71413240 | 57514091 | 57470913 | 99.92 | 49486800 | 86.11 |
|  | Uninfected 10-4*^b^* | 76784739 | 64675258 | 64667976 | 99.99 | 55076745 | 85.17 |
|  |  |  |  |  |  |  |  |

*^a^* Sz, schizont; Hz, hypnozoite; GFP-neg, GFP-negative; ID, identification; expr., expressed.

*^b^* Samples excluded. See Additional file 2 for more details.

*^c^* Number of reads that mapped to either the parasite or the host genome.

*^d^* Number of reads that mapped to the host genome.

*^e^* (# host reads / # mapped reads) × 100.

*^f^* Number of reads that mapped to host exons.

*^g^* (# expressed host reads / # host reads) × 100.
