# Supplementary material for: Transcriptional profiling of hepatocytes infected with the replicative form of the malaria parasite Plasmodium cynomolgi
Source: Malar J. 2022 Dec 23;21:393. doi: 10.1186/s12936-022-04411-3 (PMC9789591; doi:10.1186/s12936-022-04411-3)
Supplement: Supplementary file 2 — Additional file 2. Samples used in this study. Additional file table. [file 12936_2022_4411_MOESM2_ESM.docx]

**Additional file 2.** Samples used in this study*^a^*

| Infection ID | Sample ID | Sample group | Harvesting day p.i. | Hepatocyte*^b^* | Expressed host reads | Comments |
| --- | --- | --- | --- | --- | --- | --- |
|  |  |  |  |  |  |  |
| PAC.27.F1 | Sz 9-1 | GFP-high | 9 | Fresh | 17765194 |  |
|  | Hz 9-1 | GFP-low | 9 | Fresh | 22174914 | Excluded*^c^* |
|  | Negative 9-1 | GFP-neg | 9 | Fresh | 36748132 |  |
|  | Uninfected 9-1 | Uninfected | 9 | Fresh | 39888812 |  |
|  | Sz 10-1 | GFP-high | 10 | Fresh | 17334498 |  |
|  | Hz 10-1 | GFP-low | 10 | Fresh | 39385752 | Excluded*^c^* |
|  | Negative 10-1 | GFP-neg | 10 | Fresh | 36884621 |  |
|  | Uninfected 10-1 | Uninfected | 10 | Fresh | 40090399 |  |
|  |  |  |  |  |  |  |
| PAC.27.F2 | Sz 9-2 | GFP-high | 9 | Fresh | 11200792 |  |
|  | Hz 9-2 | GFP-low | 9 | Fresh | 42164869 | Excluded*^c^* |
|  | Negative 9-2 | GFP-neg | 9 | Fresh | 43192038 |  |
|  | Uninfected 9-2 | Uninfected | 9 | Fresh | 40977430 |  |
|  | Sz 10-2 | GFP-high | 10 | Fresh | 12891304 |  |
|  | Hz 10-2 | GFP-low | 10 | Fresh | 60855237 | Excluded*^c^* |
|  | Negative 10-2 | GFP-neg | 10 | Fresh | 44481305 |  |
|  | Uninfected 10-2 | Uninfected | 10 | Fresh | 31021867 |  |
|  |  |  |  |  |  |  |
| PAC.30.F2 | Sz 9-3 | GFP-high | 9 | Fresh | 242659 | Excluded*^d^* |
|  | Hz 9-3 | GFP-low | 9 | Fresh | 1970549 | Excluded*^c,d^* |
|  | Negative 9-3 | GFP-neg | 9 | Fresh | 48571581 |  |
|  | Uninfected 9-3 | Uninfected | 9 | Fresh | 54537088 |  |
|  | Sz 10-3 | GFP-high | 10 | Fresh | 12764026 |  |
|  | Hz 10-3 | GFP-low | 10 | Fresh | 46512265 | Excluded*^c^* |
|  | Negative 10-3 | GFP-neg | 10 | Fresh | 51421 | Excluded*^d^* |
|  | Uninfected 10-3 | Uninfected | 10 | Fresh | 49887419 |  |
|  |  |  |  |  |  |  |
| PAC.31.F1.F2 | Sz 10-4 | GFP-high | 10 | Frozen | 38048997 | Excluded*^e^* |
|  | Hz 10-4 | GFP-low | 10 | Frozen | 57456052 | Excluded*^c,e^* |
|  | Negative 10-4 | GFP-neg | 10 | Frozen | 49486800 | Excluded*^e^* |
|  | Uninfected 10-4 | Uninfected | 10 | Frozen | 55076745 | Excluded*^e^* |
|  |  |  |  |  |  |  |

*^a^* Sz, schizont; Hz, hypnozoite; GFP-neg, GFP-negative; p.i., post-infection; ID, identification

*^b^* All hepatocytes were isolated from rhesus macaques (*Macaca mulatta*).

*^c^* GFP-low samples were previously shown to be contaminated with uninfected cells as well as with schizonts or released merozoite transcripts at these timepoints (Bertschi et al., 2018), and were excluded.

*^d^* Samples associated with a level of expressed host reads below our quality control threshold of 2 x 10^7^ reads were excluded.

*^e^* Samples obtained from experiments conducted using frozen stocks of primary hepatocytes were excluded.
