# Supplementary figures and images for: Transcriptional profiling of hepatocytes infected with the replicative form of the malaria parasite Plasmodium cynomolgi
Source: Malar J. 2022 Dec 23;21:393. doi: 10.1186/s12936-022-04411-3 (PMC9789591; doi:10.1186/s12936-022-04411-3)

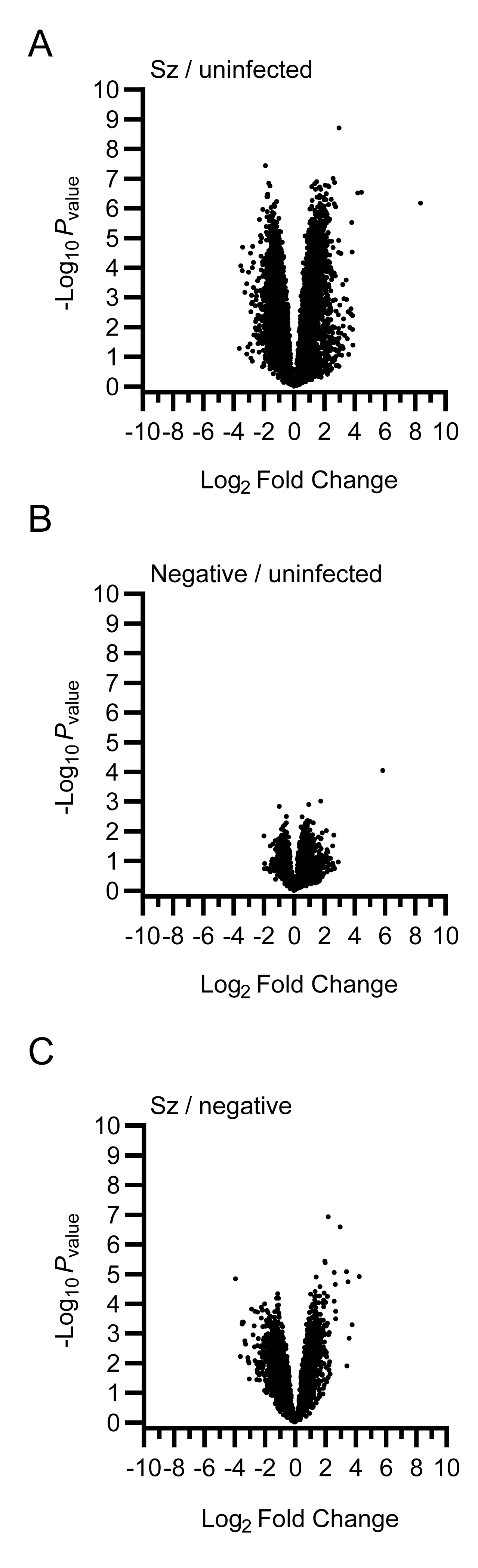

Supplement: Supplementary file 4 — Additional file 4. Volcano plots showing mean log2 fold changes and − log10 Pvalues for schizont-infected vs. uninfected samples (A), uninfected bystander (negative) cells vs uninfected samples (B) and schizont-infected vs. negative cells (C). Additional file figure. [file 12936_2022_4411_MOESM4_ESM.tif]

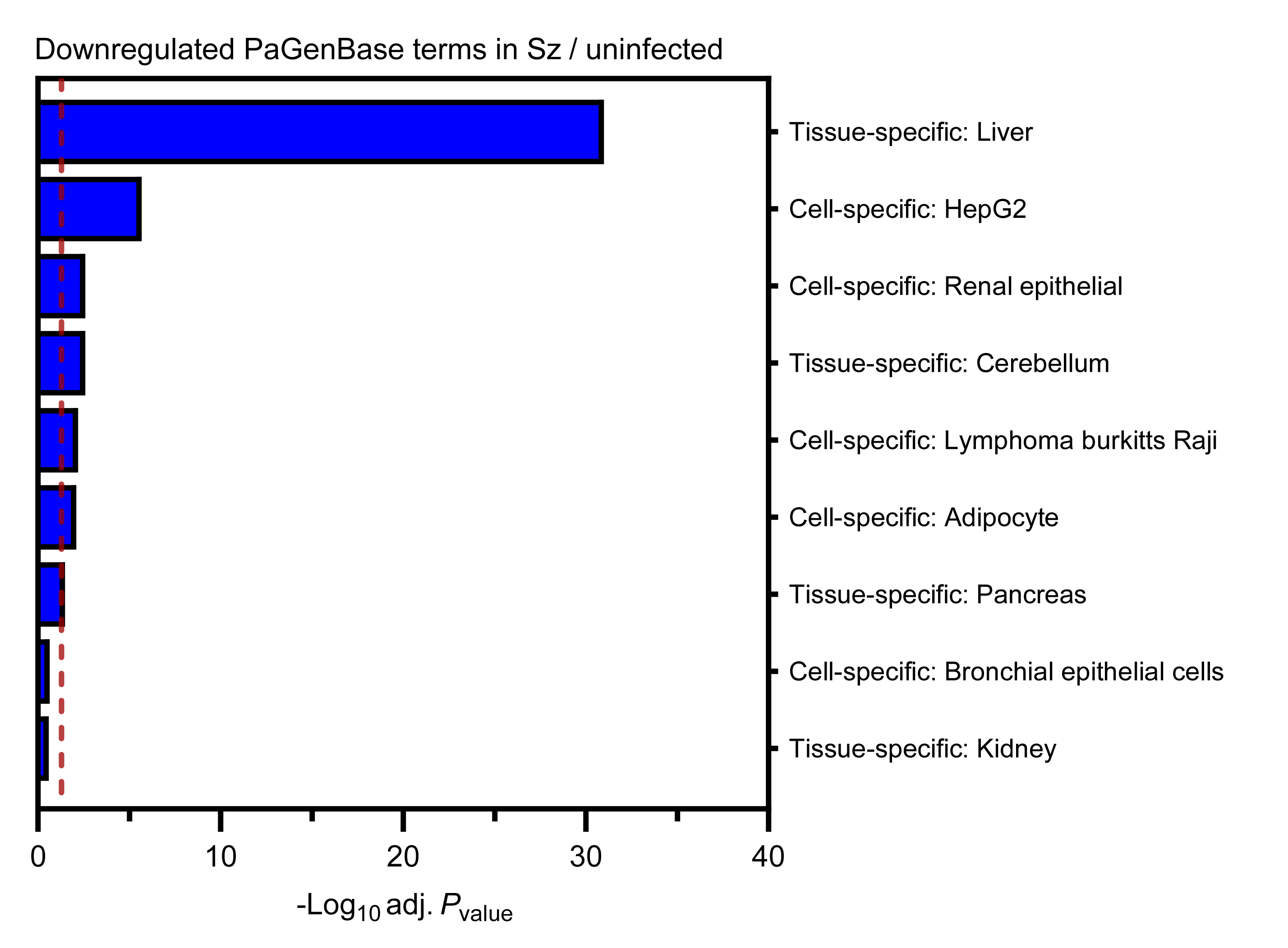

Supplement: Supplementary file 10 — Additional file 10. Most enriched PaGenBase (Pattern Gene Database) terms for genes significantly downregulated in primary rhesus macaque hepatocytes infected with schizonts in comparison to uninfected samples. The vertical dotted red line indicates adjusted Pvalues less than 0.05 (or − log10 (adj. Pvalue) greater than 1.3). This analysis was performed with Metascape. Additional figure associated with Fig. 3. [file 12936_2022_4411_MOESM10_ESM.tif]

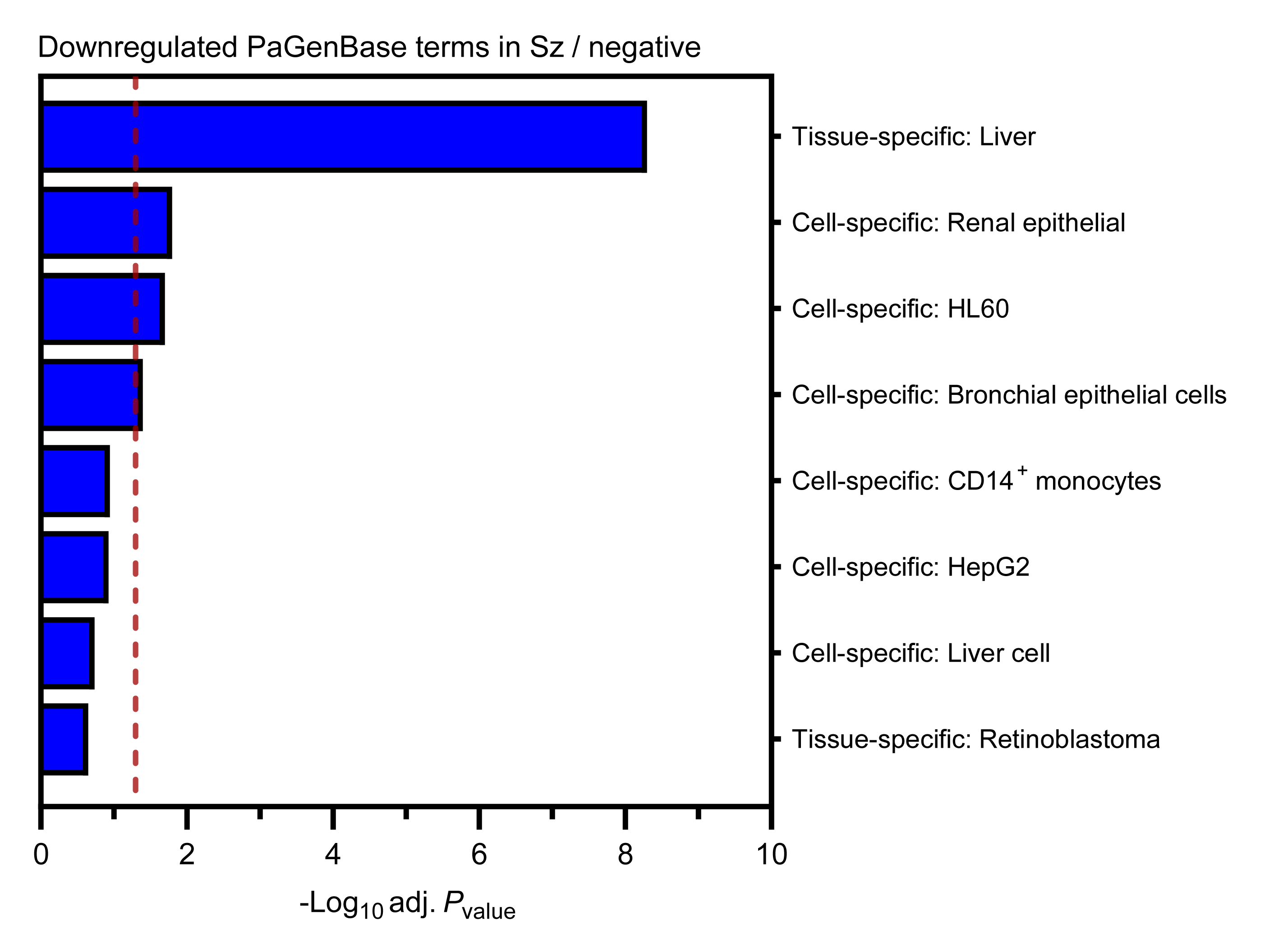

Supplement: Supplementary file 12 — Additional file 12. Most enriched PaGenBase (Pattern Gene Database) terms for genes significantly downregulated in primary rhesus macaque hepatocytes infected with schizonts in comparison to uninfected bystander (negative) cells. The vertical dotted red line indicates adjusted Pvalues less than 0.05 (or − log10 (adj. Pvalue) greater than 1.3). This analysis was performed with Metascape. Supplemental figure associated with Fig. 4. [file 12936_2022_4411_MOESM12_ESM.tif]

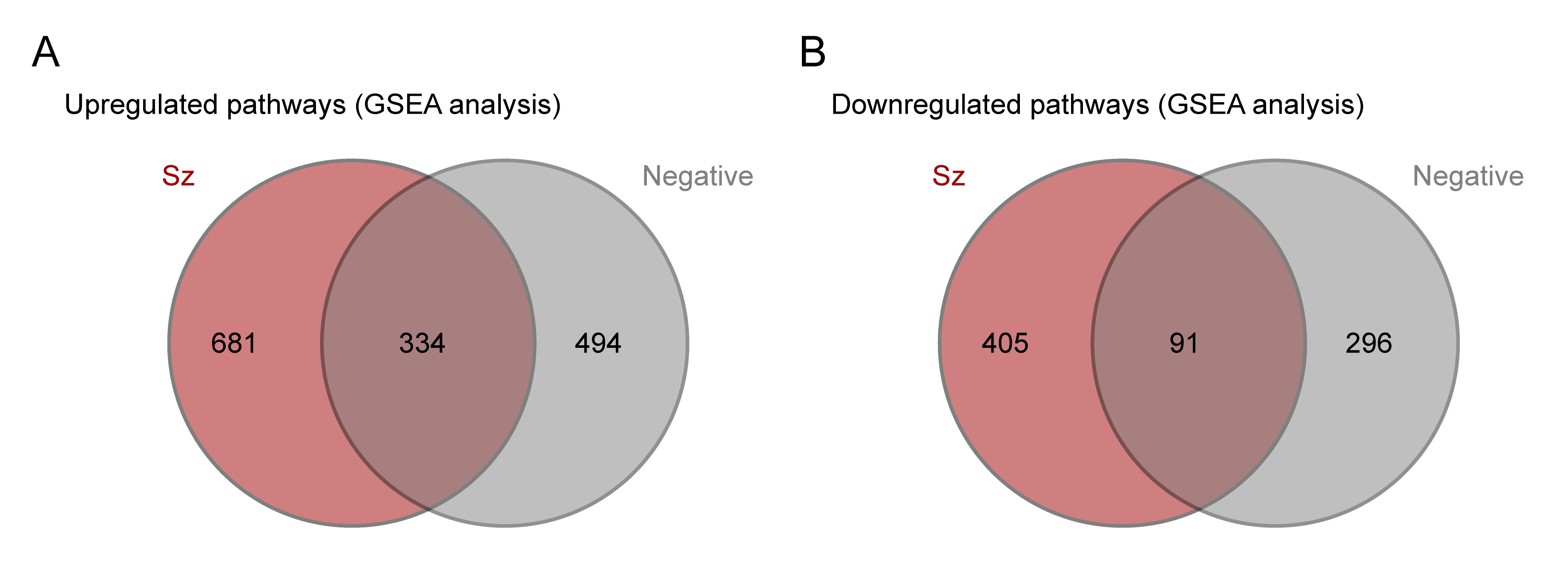

Supplement: Supplementary file 16 — Additional file 16. Venn diagram analyses comparing upregulated (A) and downregulated (B) gene sets / pathways from GSEA analyses in schizont-infected vs. uninfected samples and uninfected bystander (negative) cells vs. uninfected samples. All gene sets from the MSigDB collections were considered for these analyses. Additional file figure. [file 12936_2022_4411_MOESM16_ESM.tif]

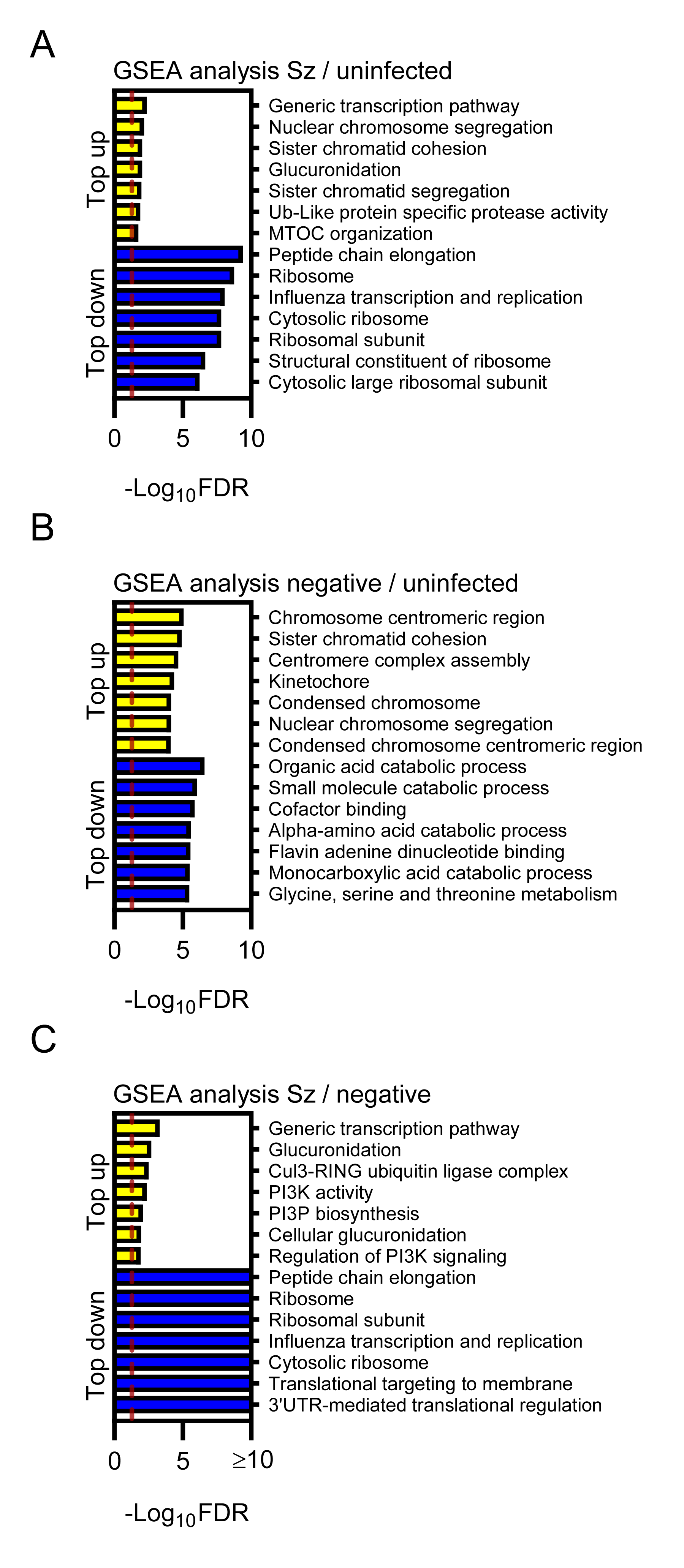

Supplement: Supplementary file 17 — Additional file 17. Most significantly enriched gene sets from GSEAs for the upregulated (yellow) and downregulated (blue) transcripts in schizont-infected vs. uninfected samples (A), uninfected bystander (negative) cells vs. uninfected samples (B) and schizont-infected vs. negative cells (C). Only gene sets from the Gene Ontology (GO), the KEGG pathway and the Reactome pathway databases were considered. Some annotation terms are abbreviated or modified for a purpose of presentation. Additional file figure. [file 12936_2022_4411_MOESM17_ESM.tif]

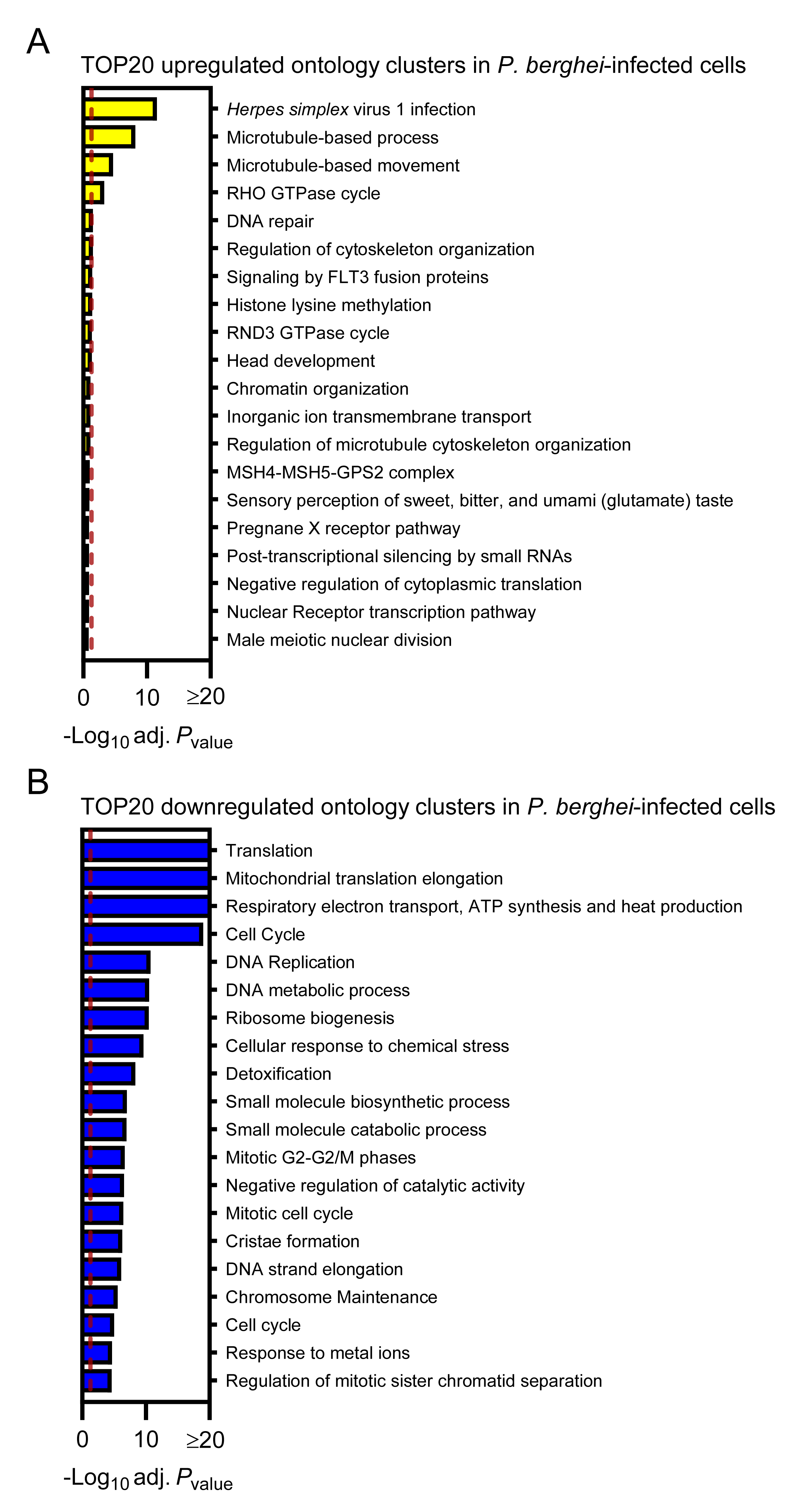

Supplement: Supplementary file 18 — Additional file 18. Re-analysis of the host response to P. berghei liver stage schizonts. Most enriched ontology clusters for genes significantly upregulated (A) and downregulated (B) in cells infected with P. berghei liver stage schizonts. Dotted red lines indicate adjusted Pvalues less than 0.05 (or − log10 (adj. Pvalue) greater than 1.3). Some annotation terms are abbreviated or modified for a purpose of presentation. This analysis was performed with Metascape. Additional file figure. [file 12936_2022_4411_MOESM18_ESM.tif]

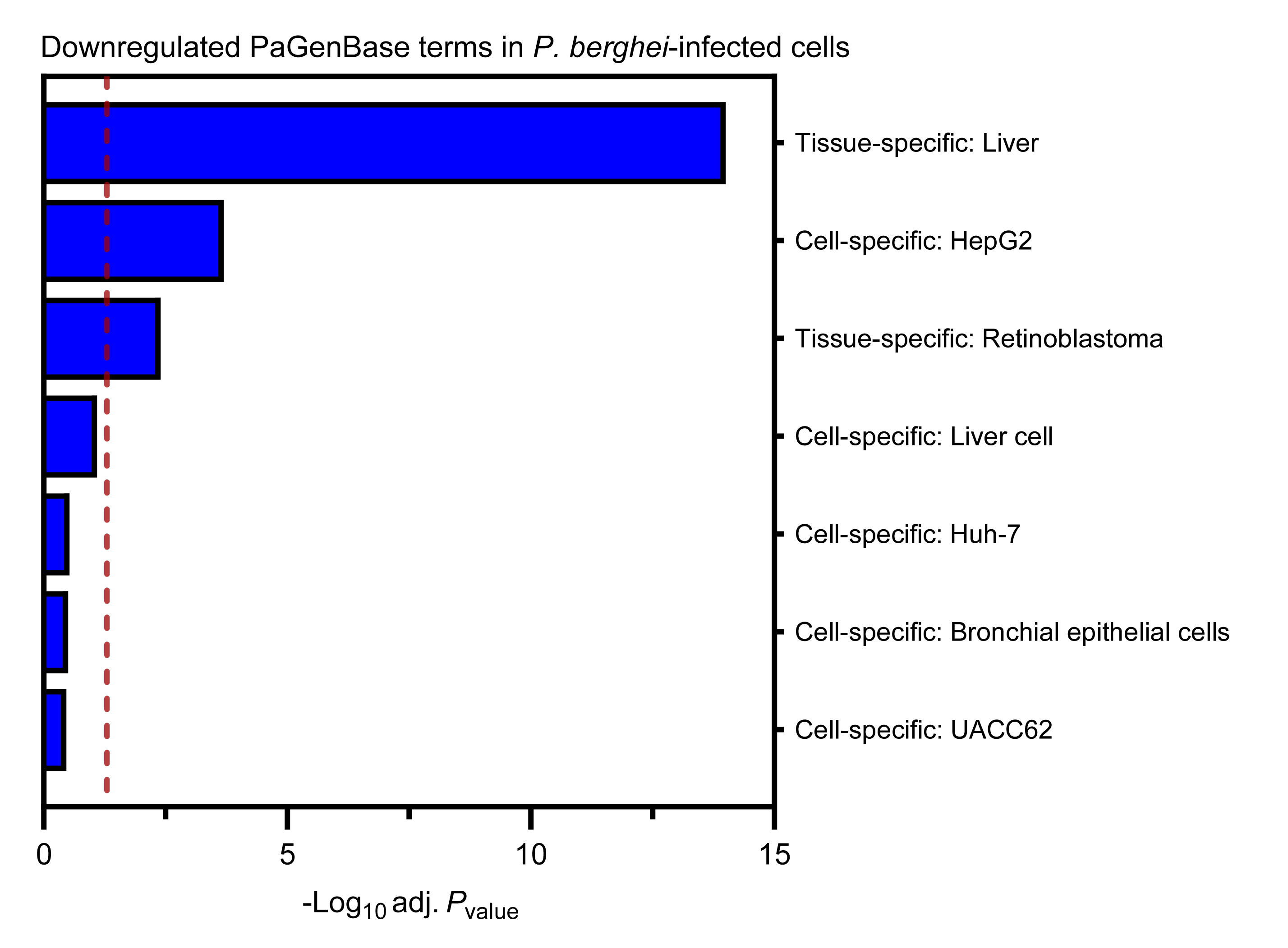

Supplement: Supplementary file 19 — Additional file19. Re-analysis of the host response to P. berghei liver stage schizonts. Most enriched PaGenBase (Pattern Gene Database) terms for genes significantly downregulated in cells infected with P. berghei liver stage schizonts. The vertical dotted red line indicates adjusted Pvalues less than 0.05 (or − log10 (adj. Pvalue) greater than 1.3). This analysis was performed with Metascape. Additional file figure. [file 12936_2022_4411_MOESM19_ESM.tif]
