# Supplementary material for: Transcriptional profiling of hepatocytes infected with the replicative form of the malaria parasite Plasmodium cynomolgi
Source: Malar J. 2022 Dec 23;21:393. doi: 10.1186/s12936-022-04411-3 (PMC9789591; doi:10.1186/s12936-022-04411-3)
Supplement: Supplementary file 5 — Additional file 5. Most up- and down-regulated genes in primary rhesus macaque hepatocytes infected with P. cynomolgi schizonts in comparison to uninfected samples. Supplemental table associated with Fig. 1. [file 12936_2022_4411_MOESM5_ESM.docx]

**Additional file 5.** Most up- and down-regulated genes in hepatocytes infected with *P. cynomolgi* schizonts in comparison to uninfected samples *^a^*

| Gene symbol | Gene description | Sz / uninfected | | Negative / uninfected | |
| --- | --- | --- | --- | --- | --- |
|  |  | Fold change | Adj. *P_value_* | Fold change | Adj. *P_value_* |
|  |  |  |  |  |  |
| UPREGULATED GENES | | | | | |
| *CXCL9* | C-X-C motif chemokine ligand 9 | 327.62 | < 0.05 | 58.00 | NS |
| *ADH7* | Alcohol dehydrogenase 7 (class IV), µ or σ polypeptide | 22.10 | < 0.05 | 2.08 | NS |
| *RANBP3L* | RAN binding protein 3 like | 18.64 | < 0.05 | -1.16 | NS |
| *DOCK10* | Dedicator of cytokinesis 10 | 14.62 | < 0.05 | 3.63 | NS |
| *MAP2* | Microtubule associated protein 2 | 14.58 | < 0.05 | 2.42 | NS |
| *CXCL11* | C-X-C motif chemokine ligand 11 | 14.24 | < 0.05 | 4.38 | NS |
| *RGS1* | Regulator of G protein signaling 1 | 14.14 | < 0.05 | 2.33 | NS |
| *RGS18* | Regulator of G protein signaling 18 | 13.51 | < 0.05 | 2.65 | NS |
| *IDO1* | Indoleamine 2,3-dioxygenase 1 | 13.40 | < 0.05 | 3.89 | NS |
| *GPR65* | G protein-coupled receptor 65 | 12.89 | < 0.05 | 3.84 | NS |
| *DOCK11* | Dedicator of cytokinesis 11 | 11.91 | < 0.05 | 2.37 | NS |
| *MXRA5* | Matrix remodeling associated 5 | 11.50 | < 0.05 | 2.49 | NS |
| *TDRD6* | Tudor domain containing 6 | 11.07 | < 0.05 | 2.83 | NS |
| *DLG2* | Discs large MAGUK scaffold protein 2 | 10.79 | < 0.05 | -1.76 | NS |
| *SYTL5* | Synaptotagmin like 5 | 10.22 | < 0.05 | 3.29 | NS |
| *SLC12A5* | Solute carrier family 12 member 5 | 9.86 | < 0.05 | 1.66 | NS |
| *MRC1* | Mannose receptor C-type 1 | 9.85 | < 0.05 | 2.15 | NS |
| *CLIC2* | Chloride intracellular channel 2 | 9.70 | < 0.05 | 3.22 | NS |
| *RGS2* | Regulator of G protein signaling 2 | 9.54 | < 0.05 | 2.39 | NS |
| *MYBL1* | MYB proto-oncogene like 1 | 9.40 | < 0.05 | 3.14 | NS |
| *CCL19* | C-C motif chemokine ligand 19 | 8.61 | < 0.05 | 1.80 | NS |
| *DKK3* | Dickkopf WNT signaling pathway inhibitor 3 | 8.56 | < 0.05 | 1.27 | NS |
| *SIRPB2* | Signal regulatory protein beta 2 | 8.55 | < 0.05 | 2.73 | NS |
| *SLC17A8* | Solute carrier family 17 member 8 | 8.19 | < 0.05 | 1.10 | NS |
| *ACKR4* | Atypical chemokine receptor 4 | 7.88 | < 0.05 | -1.04 | NS |
|  |  |  |  |  |  |
| DOWNREGULATED GENES | | | | | |
| *HOXB9* | Homeobox B9 | -11.47 | < 0.05 | -1.31 | NS |
| *RNF165* | Ring finger protein 165 | -10.61 | < 0.05 | -1.24 | NS |
| *LGALS7* | Galectin 7 | -10.61 | < 0.05 | -1.11 | NS |
| *C1orf210* | Chromosome 1 open reading frame 210 | -9.45 | < 0.05 | -1.20 | NS |
| *RHBG* | Rh family B glycoprotein | -8.76 | < 0.05 | -1.62 | NS |
| *RND2* | Rho family GTPase 2 | -8.01 | < 0.05 | -1.26 | NS |
| *CCDC103* | Coiled-coil domain containing 103 | -7.37 | < 0.05 | -1.12 | NS |
| *CLIP2* | CAP-Gly domain containing linker protein 2 | -7.25 | < 0.05 | -1.30 | NS |
| *NPB* | Neuropeptide B | -7.02 | < 0.05 | 1.04 | NS |
| *STAC* | SH3 and cysteine rich domain | -7.00 | < 0.05 | -2.16 | NS |
| *STRA6* | Signaling receptor and transporter of retinol STRA6 | -6.80 | < 0.05 | -1.36 | NS |
| *VILL* | Villin like | -6.75 | < 0.05 | -1.53 | NS |
| *APOE* | Apolipoprotein E | -6.66 | < 0.05 | -1.73 | NS |
| *SLC25A47* | Solute carrier family 25 member 47 | -6.49 | < 0.05 | -1.64 | NS |
| *H4C3* | H4 clustered histone 3 | -6.43 | < 0.05 | 1.15 | NS |
| *FBLN1* | Fibulin 1 | -6.36 | < 0.05 | -1.31 | NS |
| *FXYD2* | FXYD domain containing ion transport regulator 2 | -6.27 | < 0.05 | -1.85 | NS |
| *FXYD1* | FXYD domain containing ion transport regulator 1 | -5.94 | < 0.05 | -1.83 | NS |
| *SOAT2* | Sterol O-acyltransferase 2 | -5.91 | < 0.05 | -2.04 | NS |
| *FXYD-6/-2* | FXYD6-FXYD2 readthrough | -5.83 | < 0.05 | -1.68 | NS |
| *LGALS4* | Galectin 4 | -5.76 | < 0.05 | -1.48 | NS |
| *AQP7* | Aquaporin 7 | -5.73 | < 0.05 | -2.34 | NS |
| *C2CD4C* | C2 calcium dependent domain containing 4C | -5.73 | < 0.05 | -1.08 | NS |
| *CPT1B* | Carnitine palmitoyltransferase 1B | -5.69 | < 0.05 | -1.47 | NS |
| *RADIL* | Rap associating with DIL domain | -5.68 | < 0.05 | -1.24 | NS |
|  |  |  |  |  |  |

*^a^* Sz, schizont; Adj. *P_value_*, adjusted *P_value_*; NS, non-significant.
