# Supplementary material for: Transcriptional profiling of hepatocytes infected with the replicative form of the malaria parasite Plasmodium cynomolgi
Source: Malar J. 2022 Dec 23;21:393. doi: 10.1186/s12936-022-04411-3 (PMC9789591; doi:10.1186/s12936-022-04411-3)
Supplement: Supplementary file 7 — Additional file 7. Most up- and down-regulated genes in primary rhesus macaque hepatocytes infected with P. cynomolgi schizonts in comparison to uninfected bystander (negative) cells. Supplemental table associated with Fig. 1. [file 12936_2022_4411_MOESM7_ESM.docx]

**Additional file 7.** Most up- and down-regulated genes in hepatocytes infected with *P. cynomolgi* schizonts in comparison to uninfected bystander (negative) cells

| Gene symbol | Gene description | Fold change |
| --- | --- | --- |
|  |  |  |
| UPREGULATED GENES | | |
| *RANBP3L* | RAN binding protein 3 like | 18.95 |
| *DLG2* | Discs large MAGUK scaffold protein 2 | 13.53 |
| *LGR5* | Leucine rich repeat containing G protein-coupled receptor 5 | 11.95 |
| *SLC17A8* | Solute carrier family 17 member 8 | 11.35 |
| *ADH7* | Alcohol dehydrogenase 7 (class IV), mu or sigma polypeptide | 10.60 |
| *ACKR4* | Atypical chemokine receptor 4 | 7.92 |
| *CCL19* | C-C motif chemokine ligand 19 | 6.42 |
| *TTN* | Titin | 6.40 |
| *MAP2* | Microtubule associated protein 2 | 6.30 |
| *CCL5* | C-C motif chemokine ligand 5 | 6.00 |
| *SLC16A12* | Solute carrier family 16 member 12 | 5.99 |
| *MINAR1* | Membrane integral NOTCH2 associated receptor 1 | 5.97 |
| *ACSBG1* | Acyl-CoA synthetase bubblegum family member 1 | 4.82 |
| *RGS1* | Regulator of G protein signaling 1 | 4.64 |
| *TMEM255A* | Transmembrane protein 255A | 4.56 |
| *ADAM21* | ADAM metallopeptidase domain 21 | 4.49 |
| *PLCE1* | Phospholipase C epsilon 1 | 4.40 |
| *GCLC* | Glutamate-cysteine ligase catalytic subunit | 4.37 |
| *LRRN3* | Leucine rich repeat neuronal 3 | 4.22 |
| *FGF7* | Fibroblast growth factor 7 | 4.20 |
| *FAM171B* | Family with sequence similarity 171 member B | 4.19 |
| *GLI1* | GLI family zinc finger 1 | 4.16 |
| *ADGRF5* | Adhesion G protein-coupled receptor F5 | 4.15 |
| *HRH4* | Histamine receptor H4 | 4.11 |
| *MEF2C* | Myocyte enhancer factor 2C | 4.03 |
|  |  |  |
| DOWNREGULATED GENES | | |
| *HOXB9* | Homeobox B9 | -15.21 |
| *H1-1* | H1.1 linker histone, cluster member | -12.22 |
| *NPB* | Neuropeptide B | -11.27 |
| *LGALS7* | Galectin 7 | -11.22 |
| *C1orf210* | Chromosome 1 open reading frame 210 | -10.77 |
| *OPRPN* | Opiorphin prepropeptide | -10.07 |
| *KLK10* | Kallikrein related peptidase 10 | -9.69 |
| *CCDC71* | Coiled-coil domain containing 71 | -8.73 |
| *WDR86* | WD repeat domain 86 | -7.39 |
| *H4C3* | H4 clustered histone 3 | -6.87 |
| *PRG2* | Proteoglycan 2, pro-eosinophil major basic protein | -6.54 |
| *RNF165* | Ring finger protein 165 | -6.43 |
| *GRAMD2A* | GRAM domain containing 2A | -6.30 |
| *H1-4* | H1.4 linker histone, cluster member | -5.61 |
| *TRIM15* | Tripartite motif containing 15 | -5.54 |
| *STAR* | Steroidogenic acute regulatory protein | -5.37 |
| *C1QTNF2* | C1q and TNF related 2 | -5.03 |
| *TMIE* | Transmembrane inner ear | -4.86 |
| *CCDC103* | Coiled-coil domain containing 103 | -4.74 |
| *STRA6* | Signaling receptor and transporter of retinol STRA6 | -4.71 |
| *CDKN2A* | Cyclin dependent kinase inhibitor 2A | -4.59 |
| *GNAT2* | G protein subunit alpha transducin 2 | -4.59 |
| *RASL11A* | RAS like family 11 member A | -4.50 |
| *MMP7* | Matrix metallopeptidase 7 | -4.48 |
| *SLPI* | Secretory leukocyte peptidase inhibitor | -4.46 |
|  |  |  |
